# Cisplatin for small cell lung cancer: Associated publications in Science Citation Index Expanded

**DOI:** 10.3892/ol.2012.1029

**Published:** 2012-11-15

**Authors:** YUH-SHAN HO, KENSUKE NAKAZAWA, SHINYA SATO, TOMOHIRO TAMURA, KOICHI KURISHIMA, HIROAKI SATOH

**Affiliations:** 1 Trend Research Centre, Asia University, Taichung 41354, Taiwan;; 2 Division of Respiratory Medicine, Institute of Clinical Medicine, University of Tsukuba, Tsukuba, Ibaraki 305-8575, Japan;; 3 Division of Respiratory Medicine, Mito Medical Center, University of Tsukuba, Mito, Ibaraki 310-0015, Japan

**Keywords:** small cell lung cancer, cisplatin, web of science, bibliometric

## Abstract

This study was conducted to explore a bibliometric approach to quantitatively assess current research trends in cisplatin-containing chemotherapy for small cell lung cancer (SCLC), using related literature in the Science Citation Index Expanded database from 1992 to 2011. Articles were analyzed by the scientific output and research performances of countries and institutions. The distribution of key words in the article title and author-selected keywords were used to evaluate research trends. It was observed that the number of articles devoted to cisplatin-containing chemotherapy for SCLC did not increase with time. The USA and Japan were the top two countries with the highest number of articles devoted to cisplatin-containing chemotherapy for SCLC. In both countries, the number of articles did not increase with time, and a decreasing trend was identified in the USA over the last 10 years. This study demonstrates trends in cisplatin-containing chemotherapy for SCLC. The clinical application of novel drugs is required for successful SCLC treatment.

## Introduction

Lung cancer remains one of the most common types of fatal malignancies (1). Despite the progress made in various therapeutic modalities, the overall outcome of patients with lung cancer remains poor and has not decreased to a satisfactory level (2,3). Small cell lung cancers (SCLCs) represent 15% of all lung cancers (4). Response rates of SCLC to chemotherapy and radiotherapy are impressive; however, relapses are frequent and the survival rate at two years does not exceed 10% in metastatic patients (5). At present, platinum-based treatments are key drugs for SCLC (6). Currently, platinum plus etoposide (7) or platinum plus irinotecan (8) are the cornerstone treatment. The state-of-the-art treatment for SCLC patients involves the platinum chemotherapy regimen in combination with early thoracic radiotherapy (5). Cisplatin and carboplatin are two of the most common types of platinum-based treatments used in SCLC chemotherapy (9). In clinical trials, cisplatin is often selected due to its strong antitumor activity, but its adverse effects include renal toxicity (10), nausea and vomiting (11). Therefore, to avoid renal toxicity, urine volumes should be monitored and large-dose infusion is mandatory in cisplatin-based chemotherapy. In clinical practice, carboplatin has been considered to be a substitute for cisplatin without any apparent loss of therapeutic efficacy since aggressive hydration is often problematic. In recent years, the treatment for non-small cell lung cancer (NSCLC) has dramatically improved due to the use of molecular target drugs (12). However, in recent decades there appears to be no significant progress in the treatment for SCLC.

Bibliometric analysis has been applied to cancer-related topics, including oncology (13), genes for cancer classification (14), geography of clinical cancer (15), otolaryngology (16), translational cancer (17), facing mortality from cancer (18), characterizing cancer information systems (19), cancer molecular epidemiology (20), gynecologic oncology (21), breast cancer (22), mesothelioma (23), Japanese lung cancer (24), public policy on cancer (25), *Helicobacter pylori*(26), human papillomavirus (27), and cancer research performance in the European Union (28).

Recently, word cluster analyses based on words in article titles, abstracts and author keywords, were used for evaluation of research trends (29–31). Cluster analyses have also been applied in the research of Parkinson’s disease (32) and stem cell research (29).

The present study was designed to determine the trends in articles devoted to cisplatin-containing chemotherapy for SCLC from 1992 to 2011. A bibliometric method was used to analyze SCLC with cisplatin-related publication trends, including international collaboration, distribution of institutes and author publications.

## Materials and methods

### Science Citation Index (SCI) Expanded

Documents used in this study were derived from the SCI-Expanded database of the Web of Science (Thomson Reuters, New York, NY, USA). According to Journal Citation Reports (JCR), in 2010 8,073 journals were indexed with citation references across 174 scientific disciplines. Documents published from 1992 to 2011 were downloaded from the database and analyzed. This study was approved by ethical committee of Mito Medical Center, University of Tsukuba, Mito-city, Japan.

### Keywords

Keywords, including ‘small cell lung cancer(s)’, ‘small cell lung carcinoma(s)’ and ‘small cell carcinoma/ cancer of the lung’, as well as ‘cisplatin’, ‘cisplatinum’ and ‘cis-platinum’, were searched in terms of topic. Three parts, including the title, abstract and author keywords, were searched based on SCI-Expanded updated 21 March 2012. Documents including ‘non-small cell lung cancer/s’, ‘nonsmall cell lung cancer/s’, ‘non-small cell lung carcinoma/s’, ‘nonsmall cell lung carcinoma/s’, ‘non-small cell lung/s’ and ‘nonsmall cell lung/s’ within the title, abstract or author keywords were not considered for analysis in this study.

### Article origin

Articles originating from England, Scotland, Northern Ireland and Wales were reclassified as being from the UK, and articles from Hong Kong published after 1997 were included in the China category. A total of 554 articles were analyzed, but no other documents were included. The impact factor of a journal was determined for each document as reported in the JCR 2010.

## Results

### Document type and language of publication

The distribution of the document type identified by the Web of Science was analyzed. Nine document types were identified of a total of 809 publications during the 20 year study period. Articles were the most frequently observed document type (554 articles; 68% of the total number of publications), followed distantly by meeting abstracts (109 articles; 13%), proceedings papers (86 articles; 11%) and reviews (39 articles; 4.8%). Not only do journal articles comprise the majority of the publications, they also include full descriptions of the research. Only 554 articles were used for further analysis, and almost all of these articles were published in English.

### Article trends

**Figure 1.**
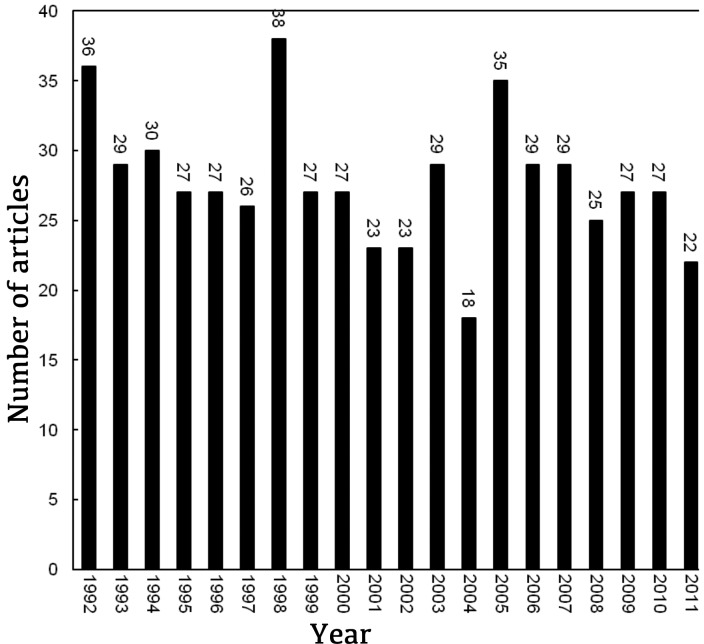
Total number of publications in Science Citation Index Expanded referring to the key words ‘small cell lung cancer’ and ‘cisplatin’ during the past 20 years.

The total number of articles in SCI-Expanded that included the search words ‘small cell lung cancer’ and ‘cisplatin’ during the last 20 years was counted. As shown in Fig. 1, the number of articles devoted to cisplatin-containing chemotherapy for SCLC did not increase over time. The 554 articles originated from 42 countries; 490 (88%) of these were independent articles and 64 (12%) were internationally collaborative articles. The collaboration rate was similar to that of other medical related areas. For example, the collaboration rate accounted for 5.3 and 5.9% of Patent Ductus Arteriosus treatment with drug and surgery research, respectively, 11% of asthma in children (33), 12% of stem cell (29), 14% of acupuncture (34), 18% of *Helicobacter pylori*(26), 18% of Parkinson’s disease (32) and 22% of human papillomavirus research (27).

**Figure 2.**
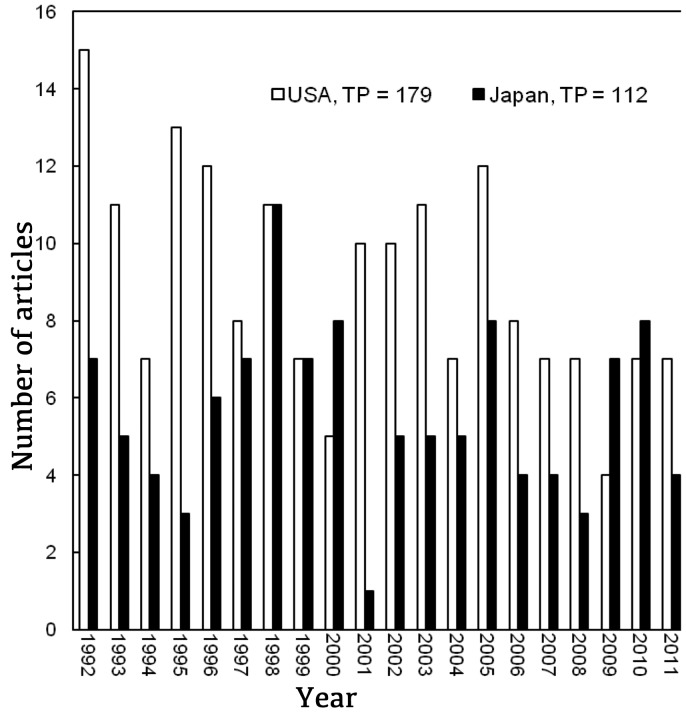
Comparison of trends between the number of articles devoted to cisplatin-containing chemotherapy for small cell lung cancer in USA and Japan. TP, total number of articles.

Table I shows the top 10 countries ranked by the number of total articles with the five indicators, including total number of articles, single country, international collaborative, first author and corresponding author articles (35). In the majority of cases, the first author contributed the greatest; however, the corresponding author obviously increased the author’s credit for contributions to the study (36). The designation of corresponding author is very important since they supervise the planning and execution of the study, as well as the writing of the paper (37). The seven major industrial countries (G7), Canada, France, Germany, Italy, Japan, UK, and USA, ranked the top seven of world publications. Similar results were also identified in the research of Parkinson’s disease (32) and stem cell research (29). Japan was ranked second in the world for the research of *Helicobacter pylori*(26) and stem cells (29). Fig. 2 shows the comparison between the trends of the number of articles devoted to cisplatin-containing chemotherapy for SCLC in the top two productive countries, USA and Japan. The number of articles did not increase with time in both countries. Notably, a decreasing trend was identified over the last ten years in USA.

### Institutional comparisons

The contribution of different institutions was estimated using the location of the affiliation of at least one author of the published articles. The most frequent institutes from which articles originated were as follows: the National Cancer Center (Japan; 25 articles), Okayama University (Japan; 20 articles), Vanderbilt University (USA; 17 articles), University of Texas (USA; 15 articles), University of California (USA; 15 articles), Mayo Clinic (USA; 15 articles), University of Groningen Hospital (Netherlands; 12 articles), University of Colorado (USA; 12 articles), National Cancer Center East (Japan; 12 articles) and National Cancer Institute (US; 11 articles).

### Output in subject categories and journals

Based on the classification of subject categories of the JCR in 2010, the output data of articles with cisplatin for SCLC-related publications were distributed between 37 SCI subject categories. Oncology was the most common category, included in 185 journals in 2010 and accounting for 467 (84%) of all articles, followed distantly by the respiratory system (82; 15% of all articles), pharmacology and pharmacy (54; 10% of all articles), radiology, nuclear medicine and medical imaging (14; 2.5% of all articles), general and internal medicine (13; 2.3% of all articles), and biochemistry and molecular biology (11; 2.0% of all articles).

A total of 554 articles were published in 126 different SCI-Expanded journals. *Lung Cancer*, listed in the categories of oncology and respiratory system with an impact factor (IF) of 3.356, was ranked first with 59 (11%) of the 554 articles. *Journal of Clinical Oncology* (IF=18.970), listed in the category of oncology, was ranked second with 48 (8.7%) articles, followed by *Cancer* (IF=5.131) with 31 articles, *British Journal of Cancer* (IF=4.831) with 27 articles, *Cancer Chemotherapy and Pharmacology* (IF=2.759) with 22 articles, *Annals of Oncology* (IF=6.452) with 22 articles, *Clinical Cancer Research* (IF=7.338) with 21 articles, *Anticancer Research* (IF=1.656) with 20 articles and the *European Journal of Cancer* (IF=4.944) with 20 articles. Additionally, the *New England Journal of Medicine* with three published articles had the highest IF (54.386).

### Words in the title and author-selected key words

The article titles and author-selected key words provide a reasonable description with regard to the articles’ subject. Related analysis could provide further information on research trends that are of concern to researchers (29,30). Examination of author-selected key words in this study period revealed that 750 author key words were used. With the exception of searching words, ‘chemotherapy’ (in 96 articles) and ‘patients’ (in 140 articles) were the most frequently used author-selected key words and article title words, respectively. Words in the title relating to antitumor drugs other than platinum were as follows: etoposide (128 articles), topotecan (34 articles), ifosfamide (23 articles) and paclitaxel (20 articles). Author-selected key words were as follows: etoposide (49 articles), irinotecan (34 articles), topotecan (24 articles), paclitaxel (11 articles), amurubicin (9 articles), ifosfamide (7 articles), gemcitabine (6 articles), docorubicin (5 articles) and epirubicin (5 articles).

## Discussion

Lung cancer is one of the leading causes of cancer-related mortality among males and females worldwide. Although the long-term outlook for lung cancer patients has not changed significantly, there have been steady improvements over the past decade with regard to the role of chemotherapy, particularly in the treatment of NSCLC (12). Response rates of SCLC to chemotherapy and radiotherapy are impressive; however, relapses are frequent and the 2-year survival rate does not exceed 10% in metastatic patients (5). Recently, we evaluated the increased number of research articles in the field of lung cancer therapy in Japan from 1991 to 2009 (24). In these study periods, the number of research articles devoted to lung cancer research increased, and it was suggested that this increase was mainly due to the rise of NSCLC-related articles. In NSCLC, particularly in lung adenocarcinoma, molecular target drugs, including epidermal growth factor receptor tyrosine kinase inhibitor (EGFR-TKI) (12), the vascular endothelial growth factor (VEGF) targeting monoclonal antibody (12), and the echinoderm microtubule-associated protein like 4-anaplastic lymphoma kinase (EML4-ALK) inhibitor, have emerged rapidly (38). The present study confirms that the majority of the increase was due to the NSCLC-related articles. However, the number of articles devoted to cisplatin-containing chemotherapy for SCLC did not increase in Japan or USA, which are two of the top countries producing articles in this research field. In USA, the number of articles devoted to cisplatin-containing chemotherapy for SCLC had a tendency to decrease within the last ten years.

This study revealed that over the past two decades the number of articles regarding cisplatin treatment for SCLC has not increased, and the progress in SCLC treatment has not improved. It is now necessary to establish the reasons why the number of clinical studies of SCLC did not increase, and to identify the next steps to be taken, in light of this result. The analysis of author key words in the present study provides information on research trends. In this analysis, the cytotoxic drugs listed in the title words were etoposide, topotecan, ifosfamide and paclitaxel, and those listed in the author-selected key words were etoposide, irrinotecan, topotecan, paclitaxel, amurubicin, ifosfamide, gemcitabine, docorubicin and epirubicin. All of those drugs were developed up to the 1990s, and no new cytotoxic drugs appeared. It is well-known that cisplatin plus etoposide and cisplatin plus irinotecan are two of the standard first-line chemotherapy regimens for SCLC (5), and during the study period, no new regimen appeared which replaced these current methods. As second-line chemotherapy, irinotecan and topotecan have been used (39), although only amrubicin and taxane attracted attention as second-line therapeutic drugs in this study period (40). There has been no clinical application of new drugs, including molecular target drugs for SCLC; however, clinical application of these novel drugs may markedly affect the progress of SCLC treatment. There are certain promising prospects for the future. The administration of an antiemetic drug, such as aprepitant, with cisplatin-based chemotherapy is highly effective in controlling nausea. At present, the majority of SCLC patients relapse within two years if a good response was obtained from the first-line chemotherapy. There are few treatment options in second-line chemotherapy for SCLC treatment compared with NSCLC treatment, and further biological research of SCLC is required to identify pathways that may be targeted with new antitumor drugs.

Using small cell lung cancer- and cisplatin-related SCI-Expanded journal articles, we obtained significant information on SCLC research trends from 1992 to 2011. The present study provided a systematically structural image, as well as indications of the impact of various SCLC research topics. The results of this study may be of interest for medical staff who are currently undertaking clinical and basic studies, and for those who will be conducting research and studying lung cancer medicine.

In conclusion, this study provides trends in research with regard to cisplatin-containing chemotherapy for SCLC. The clinical application of novel drugs is required for successful SCLC treatment.

## Figures and Tables

**Table I.** Characteristics of the top ten productive countries.

Country	TP	TP R (%)	SP R (%)	CP R (%)	FP R (%)	RP R (%)
USA	179	1 (32)	1 (30)	1 (50)	1 (29)	1 (29)
Japan	112	2 (20)	2 (21)	4 (16)	2 (19)	2 (20)
France	44	3 (7.9)	3 (5.9)	2 (23)	3 (5.8)	3 (5.3)
Germany	37	4 (6.7)	4 (4.9)	3 (20)	4 (4.9)	4 (5.1)
Canada	30	5 (5.4)	7 (4.1)	4 (16)	6 (4.3)	7 (3.3)
Italy	28	6 (5.1)	6 (4.3)	8 (11)	5 (4.5)	6 (3.9)
UK	23	7 (4.2)	8 (3.3)	8 (11)	8 (3.6)	7 (3.3)
South Korea	23	7 (4.2)	5 (4.5)	22 (1.6)	7 (4.0)	5 (4.5)
Netherlands	22	9 (4.0)	10 (2.7)	6 (14)	9 (2.9)	11 (2.4)
Greece	17	10 (3.1)	8 (3.3)	22 (1.6)	9 (2.9)	9 (3.1)

TP, total number of articles; SP, single country articles; CP, internationally collaborative articles; FP, first author articles; RP, corresponding author articles; R, rank.
